# Grinding Temperature and Surface Integrity of Quenched Automotive Transmission Gear during the Form Grinding Process

**DOI:** 10.3390/ma15217723

**Published:** 2022-11-02

**Authors:** Xiaoyang Jiang, Ke Liu, Yong Yan, Maojun Li, Pan Gong, Hong He

**Affiliations:** 1 State Key Laboratory of Advanced Design and Manufacture for Vehicle Body, Hunan University, Changsha 410082, China; 2 Jianglu Machinery & Electronics Group Co., Ltd., Xiangtan 411100, China; 3 China North Advanced Technology Generalization Institute, Beijing 100089, China; 4 State Key Laboratory of Materials Processing and Die & Mold Technology, School of Materials Science and Engineering, Huazhong University of Science and Technology, Wuhan 430074, China

**Keywords:** gear grinding, white layer, grinding temperature, surface integrity

## Abstract

Grinding burn is an undesired defect in gear machining, and a white layer is an indication of severe burn that is detrimental to gear surface performance. In this work, the influence of grinding parameters on the thickness of the white layer during form grinding of quenched transmission gear was investigated, and the microstructure evolution and mechanism of severe burn formation were analyzed. The grinding temperature increased with the grinding depth and grinding speed, with the highest level of ~290 °C. The thickness of the white layer exceeded 100 μm when the grinding depth was 0.03 mm, and the top layer was a plastic deformation layer followed by a fine-grained martensite layer. Coarse-grained acicular martensite was found at the interface between the white layer and softened dark layer. The mechanical effect and thermal softening mainly contributed to the formation of white layer stratification. The ground surface topography showed several scratches and typical grooves; when grinding depth increased to 0.03 mm, the grinding surface roughness Sa was relatively high and reached up to ~0.60 μm, mainly owing to severe plastic deformation under grinding wheel extrusion and the thermal effect.

## 1. Introduction

With the fast development of the vehicle industry and the increasing demand for transmission gears, it is important to manufacture gears with high quality and efficiency. For heavy-duty gears, the gear blank is usually subjected to heat treatment such as carburizing, nitriding, and quenching to meet the high hardness and toughness requirements. However, the deformation caused by heat treatment reduces the dimensional accuracy of gears. Grinding is normally applied as the final process in gear manufacturing to eliminate geometric deformation generated during heat treatment and to produce components with good geometric accuracy and surface quality [1,2,3,4]. Owing to the friction/shearing between abrasive particles and workpiece material, the grinding temperature is relatively high. Grinding burn occurs easily when the grinding parameters are unreasonable, which can be defined as changes caused by heat release out of the grinding process in the surface and subsurface layer of the workpiece [5]. Grinding burn originates from tempering and thermal softening when the grinding temperature exceeds the tempering temperature during grinding, and its essence is the transformation of the metallographic phase in the surface layer [3,6]. Grinding burn is usually divided into three levels, including predominantly dark blue oxide layers, thermal softening when the grinding temperature exceeds the austenitizing temperature, and a white layer and dark tempered layer. Grinding burn can induce machining defects such as microcracks on the ground surface [7]. 

As grinding burn is highly related to the final quality of machined products, researchers carried out an analysis on the formation mechanisms of grinding burn and how to reduce the probability of it occurring. Guerrini et al. [1] found that, when the grinding temperature exceeded 450 °C, a softened dark layer caused by carbon coming out of the solution was observed below the gear surface, and the thickness of the tempered layer increased with the grinding temperature. When the temperature exceeded the austenitization temperature, a severe bright martensite layer was formed on the outermost surface. It was found that the increase in cutting depth and feed speed led to higher level of grinding burn [3]. Sun et al. [8] reported that a white layer structure was generated on the surface layer after grinding hardening and its composition was analyzed, while the formation mechanism of the white layer and its connection with grinding parameters have not been further discussed. As the grinding depth increased, greater grinding energy was required to remove materials [9]. This indicated that more energy was converted into heat and significantly affected the workpiece surface. Su et al. [10] reported that the increase in grinding depth increased the thickness of the ablated layer, while an appropriate increase in feed speed and decrease in grinding speed could reduce the grinding temperature. Zhou et al. [11] found that, when the abrasive particles cut or ploughed across the rail, the grinding temperature decreased with the increasing forward speed, resulting in a small thickness of the white layer. Jamshidi et al. [5] defined surface burn as a chemical reaction between oxygen and workpiece material and proposed a burn model that comprehensively considered maximum temperature and exposure time to predict oxide layer thickness. Jermolajev et al. [12] established a time–temperature diagram showing surface layer modification for profile gear grinding based on local contact zone temperature measurements and used the tempering time to explain workpiece surface burn rather than contact time, as the grinding temperature could not drop below the tempering temperature instantaneously when the grinding wheel moved away.

Based on the comprehensive literature review, it was found that previous studies mainly worked on the theoretical models of grinding burn during surface grinding and the prediction of temperature thresholds for generating grinding burn. Several studies focused on the relationship between burn formation during gear grinding and grinding parameters. In this paper, according to the variation in grinding temperature and thickness of the ablation layer under different grinding parameters, the influence of grinding speed and depth on grinding burn was analyzed. The change in microhardness within subsurface layers, the microstructure and morphology, as well as the surface roughness were comprehensively investigated. The result of this study can mostly likely provide benchmarks for improving both the quality and efficiency during gear grinding in vehicle industries.

## 2. Experimental Setup and Procedure

### 2.1. Workpiece Material

A gear made of 20Cr2Ni4A steel was selected as the workpiece in this research. It underwent a series of heat treatments including normalizing, carburizing, quenching, and tempering. The corresponding chemical composition is shown in Table 1. Good toughness and high strength are required when 20Cr2Ni4A steel is used to manufacture heavy-duty gears, and it needs to be carburized and quenched to improve the hardness of its surface layer. The initial workpiece was cut into several segments for grinding trials with different grinding parameters, and the size of the samples was designed to be clamped and ground easily. 

### 2.2. Experimental Setup

**Figure 1:**
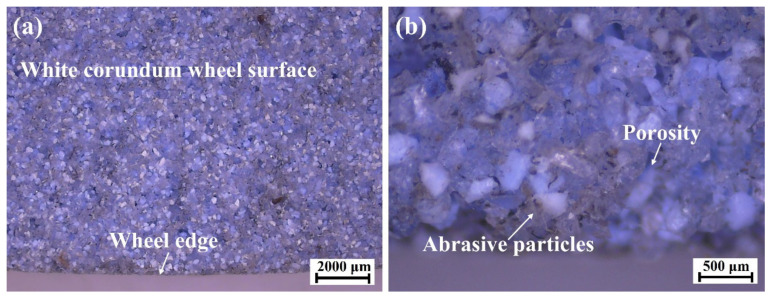
Topography of the grinding wheel surface observed by microscope with (**a**) low and (**b**) high magnification.

A three-axis automatic hydraulic high-precision grinding machine (DY-510ASM) was used for gear grinding experimental trials, which was driven by an O05-63B0 three-phase asynchronous motor. The maximum rotation speed of the grinder was 4000 r/min with a rated power of 7.5 kW. An alumina forming grinding wheel with #80 abrasive mesh and a diameter of 350 mm was used, and the grinding wheel was dressed by a diamond wheel dresser before each set of experiments. The wheel topography was observed with a high-definition charge-coupled device (HD CCD) microscope, as shown in Figure 1; white corundum abrasive grains were evenly bonded to the surface and sharp cutting edges of abrasives could be observed. 

Before grinding, the runout on both sides of the tooth surface along the feed direction was limited within 0.001 mm by a dial indicator to ensure machining accuracy. The gear sample was clamped by the fixture on the perpendicular direction of feed speed. Considering the setting of gear machining parameters in industrial application, the total grinding thickness of one side was set at 0.24 mm, the feed speed and the grinding speed were selected with three levels, and the grinding depth for each pass was set to 0.02 mm and 0.03 mm. The specific grinding parameters are shown in Table 2.

### 2.3. Measuring Equipment

**Figure 2:**
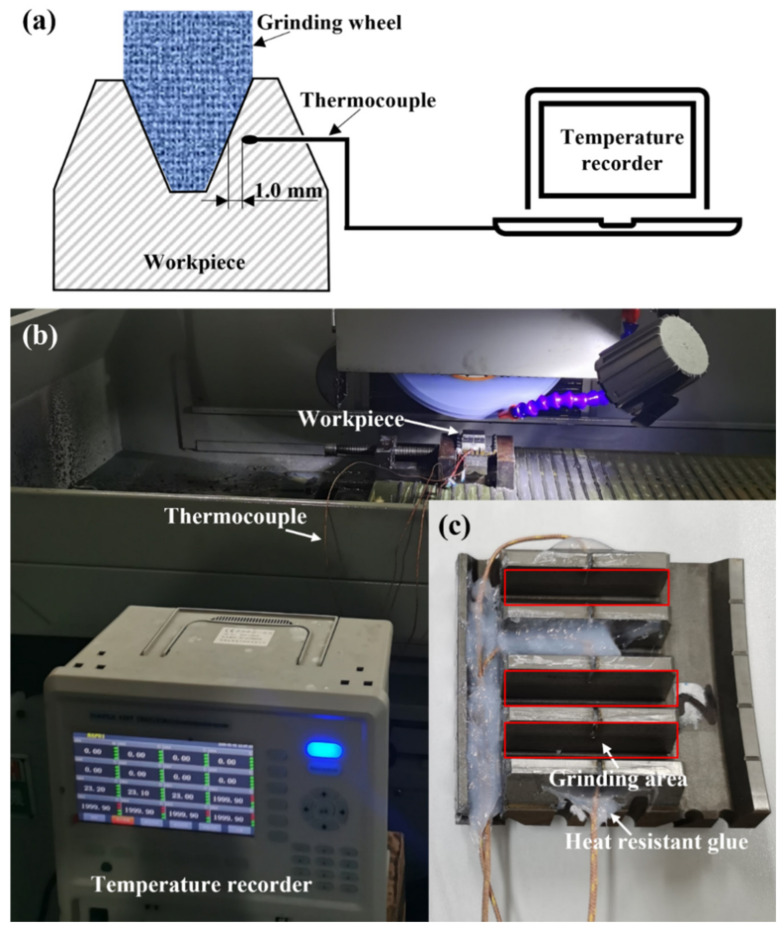
(**a**) Configuration of embedded thermocouples, (**b**) experimental setup of grinding temperature measurement, and (**c**) corresponding gear sample.

The temperature signals were captured by a GG-K-30-1000-CZ type thermocouple produced by KAIPUSEN when the grinding wheel passed the workpiece material surface. For each gear sample, a narrow slot with a width of 1.0 mm was cut from the tooth tip to the tooth root along the middle of tooth width to accommodate the thermocouple sensing tip. As shown in Figure 2a, a thermocouple was inserted into the narrow slot on the workpiece to measure the grinding temperature. This method was generally used to record temperature variation with good accuracy [14,15]. The thermocouple sensing tips were located close to the grinding surface, and the narrow slots were sealed with waterproof and heat-resistant glue, which could prevent the cooling liquid from affecting the measurement results. Figure 2b shows the experimental setup of grinding temperature measurement; the gear sample selected for the grinding temperature measurement is shown in Figure 2c.

The gear teeth ground by different parameters were cut using a wire electrical discharge machine and embedded in resin for polishing to observe the microstructure of the ground surface layer. A high-resolution scanning electron microscope (SEM, TESCAN MIRA4 LMH) was used to observe the ground surface morphology. Microhardness at the subsurface was measured using a Vickers hardness tester with a load of 1000 g and intent time of 10 s, and each measurement was set with 100 μm depth intervals. An Atometrics-NA500 white light interferometer was also applied to measure the surface roughness of the ground surface.

## 3. Experimental Results and Discussion

### 3.1. Grinding Temperature

**Figure 3:**
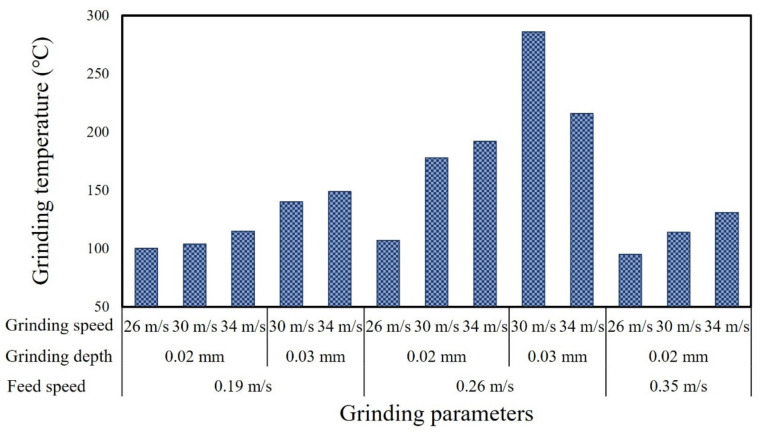
Temperature variation under different grinding parameters.

Most of the mechanical work occurring in the grinding process is converted into grinding heat, which increases the temperature of the gear tooth surface. If the thermal stress caused by the grinding process exceeds the yield strength of the workpiece material, residual tensile stress will be generated within the ground surface and it is possible to form dominant or recessive grinding cracks in the surface layer, which severely affect the gear strength and fatigue life [16]. In addition, as the contact arc surface of gear grinding is complex, the grinding temperature changes with the normal grinding depth and presents as a non-uniform form [17]. Thus, the grinding temperature is an important indicator of the workpiece heating state during the grinding process. The highest temperature during grinding under each set of parameters was recorded. Figure 3 shows the variation in grinding temperature under different grinding parameters. It is obvious that a high level of grinding parameters generally led to a relatively higher grinding temperature, with the maximum value of ~290 °C.

**Figure 4:**
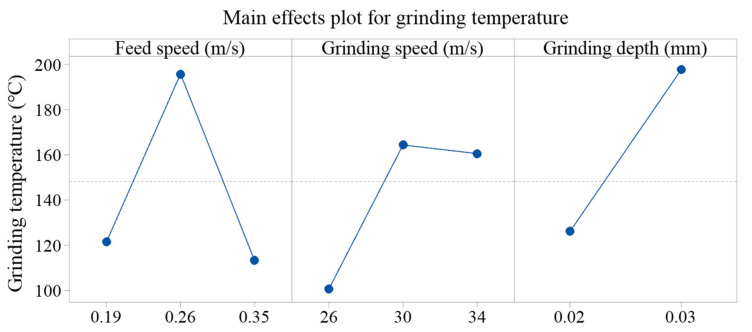
Main effects plot for grinding temperature at different parameters.

Statistical ANOVA method was applied to further analyze significant factors and the related percentage contribution ratio (PCR) for ground temperature. Figure 4 shows the detailed information concerning the main effects plots of grinding temperature related to different grinding parameters. Data from the ANOVA table confirmed that feed speed was statistically significant, affecting grinding temperature with a corresponding percentage contribution ratio of 33.7%, followed by grinding depth, with the details presented in Table 3. 

**Figure 5:**
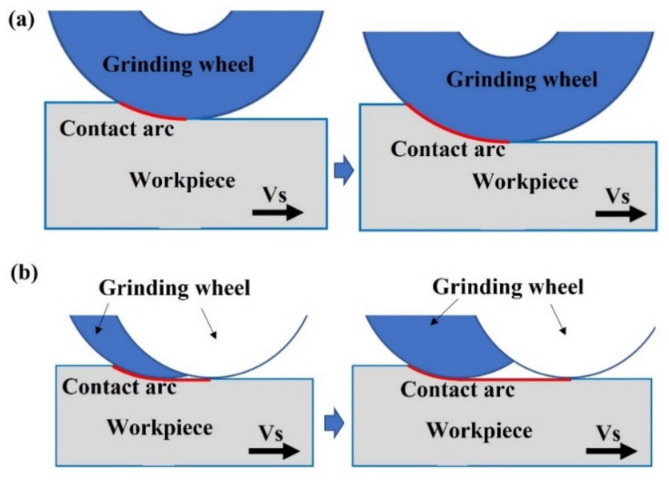
The influence of different (**a**) grinding depths and (**b**) feed speeds on contact arc length per unit time.

Generally, the increase in feed speed and grinding depth led to the increase in grinding temperature. Firstly, at a constant grinding speed, the grinding force increased with the increase in grinding depth and feed speed. It led to an increase in the total energy consumed by grinding, which indicated that more energy was converted into grinding heat [2]. The increase in grinding force also caused severe friction, which eventually led to the increase in grinding temperature. Secondly, as shown in Figure 5a, when the grinding depth increased, the actual cutting thickness increased, which not only enlarged the contact area between grinding wheel and workpiece, but also hindered the heat dissipation within the grinding area, and finally contributed to the increase in grinding temperature [18]. Figure 5b shows the influence of different feed speeds on grinding temperature. When the feed speed increased, the path traveled by the grinding wheel under per unit time was extended and more heat was generated, resulting in higher heat and grinding temperature. However, increasing the feed speed might also shorten the heat transfer time, and thus lead to a lower grinding temperature [3,19]. According to the recorded data, this kind of effect was more obvious when the feed speed increased to 0.35 m/s. With the same level of feed speed and grinding depth, the grinding temperature increased when the grinding speed increased from 30 m/s to 34 m/s, and the effect of the grinding speed was more significant when the grinding depth was set at 0.02 mm. The grinding temperature was relatively high when the grinding speed was selected as 34 m/s and the feed speed was 0.26 m/s. This was mainly because of the fact that the number of cuts per unit time increased at a higher grinding speed and, subsequently, more heat was generated [20]. The reduction in grinding temperature when increasing the grinding speed with the depth of 0.03 mm and feed of 0.26 m/s could be attributed to two aspects. One possible reason was the dimensional deformation of the gear blank during carburization and quenching processes, which might lead to uneven thermal field distribution in the grinding process. The other was that, at the depth of 0.03 mm and feed of 0.26 m/s, the scratch and ploughing effects were less significant when the grinding speed increased to 34 m/s, resulting in a decrease in the energy converted into grinding heat.

### 3.2. Characteristics of Grinding Burn

**Figure 6:**
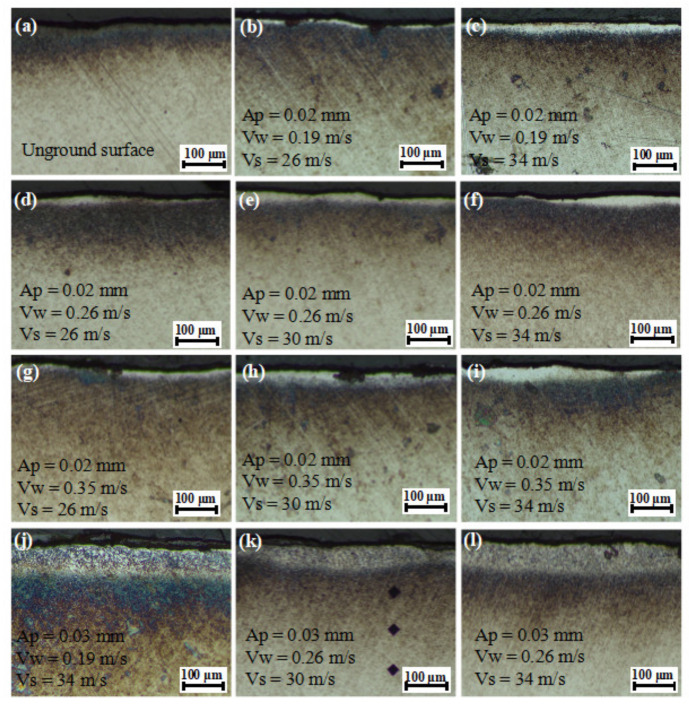
Morphology of (**a**) the unground surface layer and (**b**–**l**) the ground surface layer at different grinding parameters.

Figure 6 shows the morphology of the subsurface layer after grinding with different parameters. It was found that the bright white ablation layer generally occurred within the subsurface layer after grinding in the depth direction. The white layer was the reinforced structure of the surface material after hardening during abrasive grinding, and it was mainly composed of martensite, cementite, and residual austenite [8]. This phenomenon can be explained by the following reasons. The high temperature generated during grinding promoted the reaching of the austenitic field followed by rapid cooling, and the cooling fluid contributed to the transformation from the austenite to martensite phase [21]. White layer formation was fundamentally dependent on the thermal and mechanical effects of grinding on the workpiece material. These effects were mainly caused by the interaction of grinding tool and workpiece material and were significantly affected by grinding parameters [22].

With the grinding depth of 0.02 mm, feed speed of 0.19 m/s, and grinding speed of 26 m/s, a relatively thin white layer with a thickness of less than 5 μm was generated on the ground surface. When the grinding speed increased from 26 m/s to 34 m/s, the thickness of the white layer increased slightly. When the feed speed was 0.35 m/s and the grinding speed was over 30 m/s, the thickness of the white layer exceeded ~50 μm. This was mainly because of the fact that the exposure time was the same when the feed speed was constant, while the temperature increased with a higher grinding speed, resulting in more obvious grinding burn and a thicker white layer. The grinding burn often indicated the changes in mechanical properties caused by microstructure transformation, which ultimately led to the scrapping of the machined part [3]. At the grinding depth of 0.02 mm and the same grinding speed, the variation in white layer thickness with different feed speeds was almost consistent. Under the condition of the same grinding depth and grinding speed, the increase in feed speed led to a higher heating rate and strain rate [23]. When the grinding depth increased to 0.03 mm, a white layer with a thickness of ~100 μm was generated. A higher grinding depth resulted in a great increase in grinding area and slow dissipation of heat, which further promoted grinding burns [18]. 

**Figure 7:**
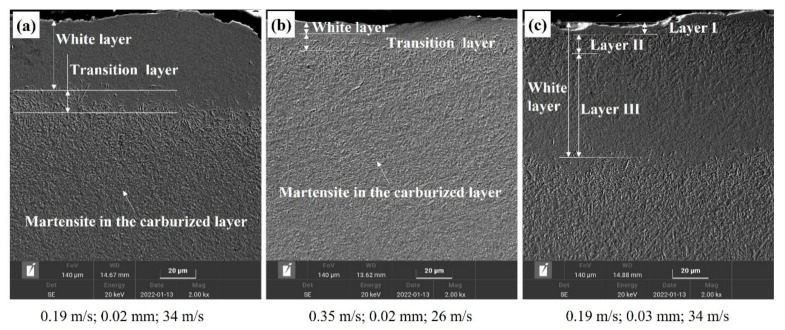
SEM morphology of the ground surface layer at the feed speed, grinding depth, and grinding speed of (**a**) 0.19 m/s, 0.02 mm, and 34 m/s, respectively; (**b**) 0.35 m/s, 0.02 mm, and 26 m/s, respectively; and (**c**) 0.19 m/s, 0.03 mm, and 34 m/s, respectively.

Figure 7 shows the SEM morphology of the ground surface layer under different grinding parameters. The effect of grinding parameters on the thickness of white layer and plastic deformation, which were most likely related to dynamic recrystallization caused by a high temperature and thermal stress during the grinding process [8]. This phenomenon caused higher hardness of the surface layer, which easily caused microcracks. Coarse-grained acicular martensite was observed at the interface between the white layer and original workpiece material, which might be the transition zone formed by the gradual weakening of thermal influence in this zone. The thickness of the white layer in Figure 7c reached ~100 μm, which could be divided into three levels. The outmost layer (layer I) was presumed to be a plastic deformation layer, and the microstructure was difficult to observe and was probably produced by abrasive particles of high-speed cutting at a high temperature. Layer II was composed of fine-grained martensite and possibly carbide particles precipitated by a high temperature. Owing to thermal and mechanical effects, the grains generated a series of dislocations and fragmentation, and finally formed the special layer after cooling [8]. In layer III, the martensite grains became coarse and retained austenite was observed, indicating that the quenching effect might be caused by a high level of the grinding temperature. In fact, when the temperature was above the austenitic transformation point and cooled rapidly, the martensitic microstructure would be generated [24]. 

**Figure 8:**
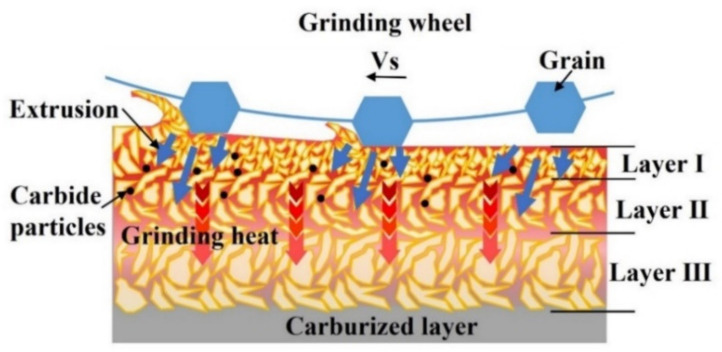
Schematic showing the forming mechanisms of the white layer during grinding of quenched gear samples.

There were two main reasons for the generation of the white layer during grinding quenched transmission gear; one was phase transformation caused by a high temperature and rapid cooling and the other one was plastic deformation caused by grinding abrasives [22]. Figure 8 shows the schematic of the formation mechanism of layer I, layer II, and layer III. When the temperature did not reach the austenite transformation point, the white layer could be formed by plastic deformation [24]. In the surface layer, owing to the strong mechanical extrusion caused by the increase in grinding depth, the material was plastically deformed and the refined grains formed layer I. Below this layer, the plastic deformation gradually decreased and, with the influence of grinding heat, a relatively obvious fine-grained martensite structure was generated, and it became the main composition of layer II. When grinding heat continued to be conducted to the subsurface, the influence of mechanical extrusion could be ignored and resulted in phase transformation during the rapid cooling process; subsequently, part of austenite transformed to martensite, forming layer III. The final properties of the surface layer were determined by the combination of work hardening caused by abrasive extrusion and heat softening caused by grinding temperature [20]. It was found that, with aggressive parameters, especially large grinding depths, undesirable grinding burn would occur on surface layer owing to the dominance of mechanical extrusion and thermal effects. The micro-structural transformation of surface layer material led to the deterioration of material properties, which ultimately caused the decrease in the surface rolling relief fatigue strength [22].

### 3.3. Microhardness and Surface Roughness Variation

**Figure 9:**
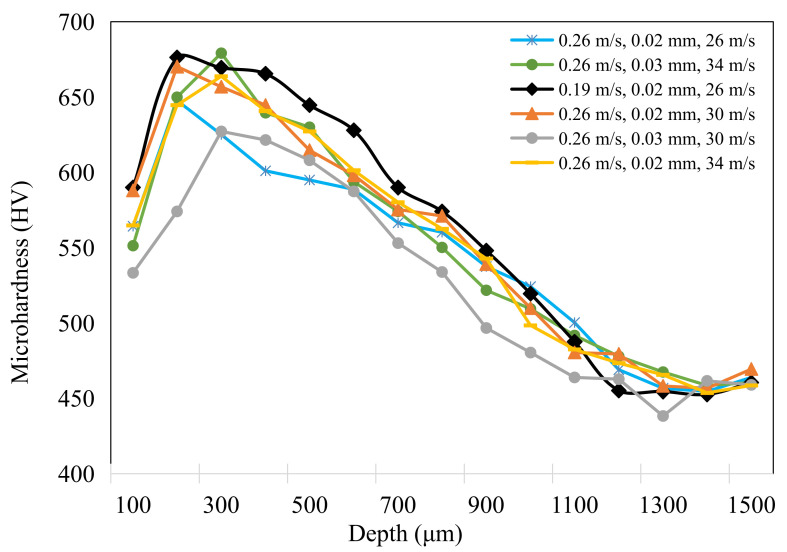
Microhardness variation within the subsurface layer when grinding quenched gear samples with different parameters.

Figure 9 shows the microhardness variation of ground surface with different grinding parameters. Before grinding, the workpiece material had a very high carbon content (≥0.35 wt.%) owing to the existence of the carburized layer, which resulted in the high hardness of surface layer, and quenching treatment led to compressive stress on the surface. The microhardness gradually decreased with the depth below surface, and finally reached the value of base material (450 HV to 460 HV) [25]. The difference in the hardness gradient after grinding could be easily observed through the variation in the microhardness curves. The hardness decreased within a 200 μm thickness owing to the influence of grinding temperature, which resulted in heat softening in this depth range [26]. The softening effect was more pronounced with a grinding depth of 0.03 mm and grinding speed of 30 m/s and 34 m/s. This was in agreement with the theory of thermal softening during the grinding process, as high temperatures were more likely to be generated at a higher grinding depth and speed [27,28]. When the grinding depth was reduced to 0.02 mm, owing to the relatively low grinding temperature, the effect of thermal softening was difficult to observe and the work hardening could be found from the hardness gradient curve. With a relatively low level of grinding parameters, the highest microhardness value reached 680 HV at the depth of 200 μm, which normally formed at the place in which the most drastic plastic deformation took place because of the removal of materials under a high grinding pressure. The occurrence of work hardening and thermal softening was related to the grinding parameters. According to the findings from Ding et al. [29], a competing process between thermal softening and work hardening occurred when the workpiece material was subjected to a high grinding temperature and large grinding pressure. In this work, aggressive grinding parameters produced a higher grinding temperature and the effect of thermal softening on the hardness gradient was obvious, while a low level of grinding parameters weakened the thermal effect and contributed to grain refinement under mechanical extrusion [30]. 

**Figure 10:**
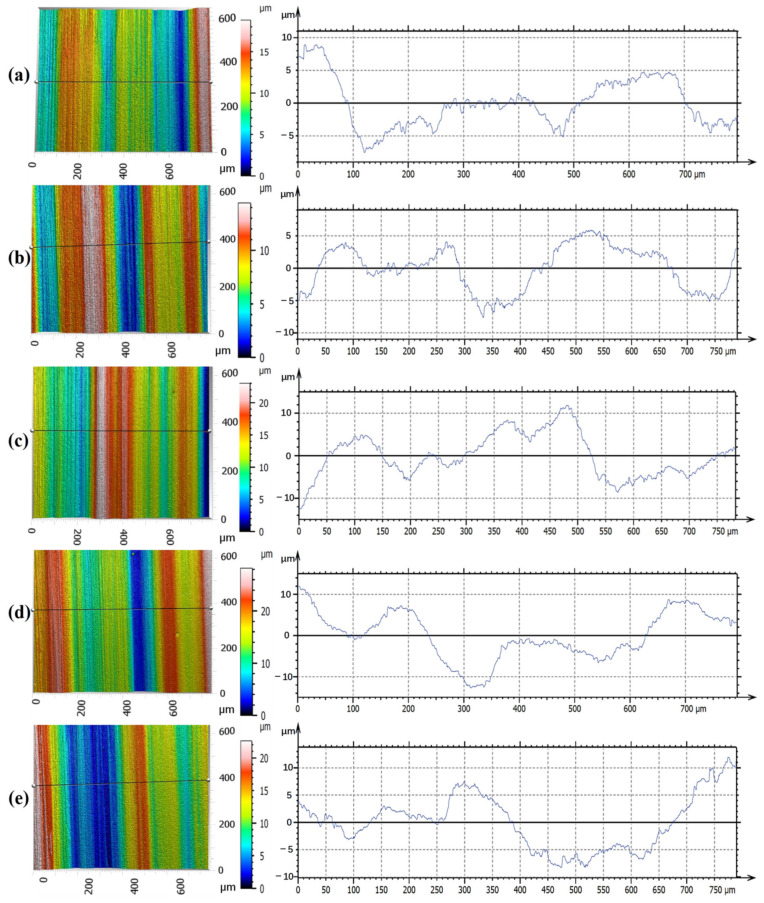
Ground surface profile under different grinding parameters (feed speed, grinding depth, and grinding speed): (**a**) 0.19 m/s, 0.02 mm, and 26 m/s, respectively; (**b**) 0.19 m/s, 0.02 mm, and 34 m/s, respectively; (**c**) 0.19 m/s, 0.03 mm, and 30 m/s, respectively; (**d**) 0.26 m/s, 0.02 mm, and 26 m/s, respectively; and (**e**) 0.35 m/s, 0.02 mm, and 34 m/s, respectively.

Figure 10 shows the ground surface profile under different grinding parameters observed via white light interference. Grooves featured by the cutting marks of a large number of abrasive grains were observed on the ground surface regardless of grinding parameters. The inherent result was determined by characteristics such as the abrasion, size, and bond type of the grinding wheel during the grinding process [20]. The corresponding surface profile was featured with peaks and valleys, especially at higher feed speeds or grinding depths. With the increased feed speed, the number of abrasive grains involved in the cutting process per unit time decreased. Therefore, the depth of grooves generally increased. With the result of a high temperature and crushing of abrasive particles, deep grooves and chip adhesion were observed, mainly owing to the fact that residual materials accumulated under the plowing effect during the grinding process [31]. 

**Figure 11:**
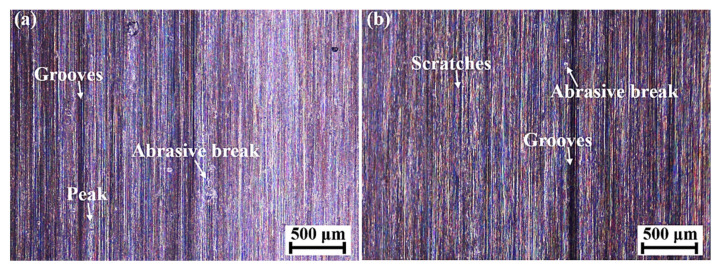
Ground surface morphology with the feed speeds, grinding depths, and grinding speeds of (**a**) 0.19 m/s, 0.02 mm, and 30 m/s, respectively, and (**b**) 0.19 m/s, 0.03 mm, and 34 m/s, respectively.

The material accumulation and deep grooves might be attributed to the factors including grain wear flat, the fractures of grains and bond bridges, together with workpiece material adhesion at high grinding forces and temperatures [26]. Figure 11 shows the topography of the ground surface with different grinding parameters. A large number of small scratches and grooves were observed. Obvious grooves were generated when the depth of scratches increased and the cross section of grooves was similar to the shapes of abrasive grains [11]. A large groove depth and small radius of curvature at the bottom of the groove could increase the coefficient of the effective stress concentration on the ground surface and increase the risk of fatigue failure [18]. It was found that particles penetrated the workpiece surface, which might be caused by the detachment and fracture of abrasive grains during the extrusion process of the grinding wheel. This defect added potential damage to the ground surface, as it not only affected surface integrity, but also probably led to surface corrosion and microcracking.

**Figure 12:**
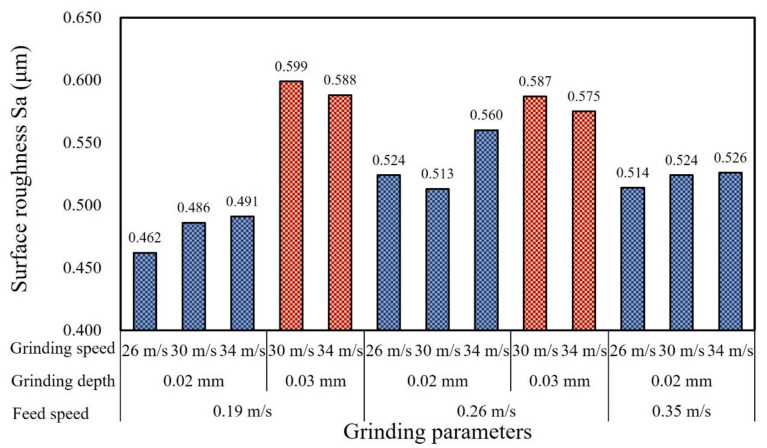
Surface roughness (Sa) variation under different grinding parameters.

**Figure 13:**
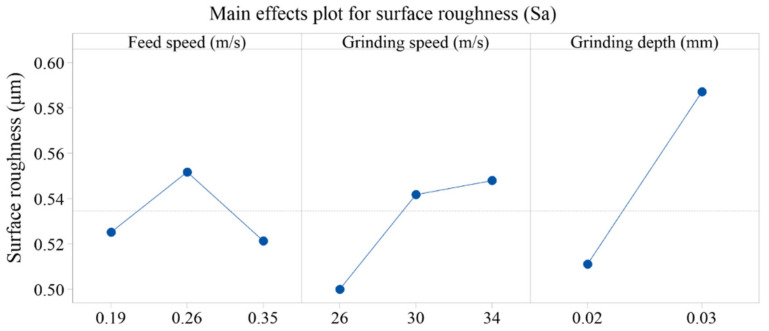
Main effects plot for surface roughness (Sa) with different parameters.

Figure 12 shows the variation in surface roughness (Sa) values of the ground surface with different grinding parameters. ANOVA was utilized to further analyze the significant factors and corresponding PCR for surface roughness (Sa). Compared with the data from ANOVA shown in Table 4, it was found that the grinding depth was statistically significant, affecting the surface roughness. Figure 13 shows the detailed information concerning the main effects plots of surface roughness related to different grinding parameters. When the grinding depth was 0.02 mm, the surface roughness gradually increased with the grinding speed and the maximum value reached 0.560 μm, which was obtained with the feed speed of 0.26 m/s and grinding speed of 34 m/s. Generally, the increase in grinding speed led to the decrease in surface roughness because it increased the number of abrasive cuts in the same period, which reduced the grinding force. However, a different trend of Sa values was observed with the grinding depth of 0.02 mm. It could be explained that the grinding temperature increased with a higher grinding speed, and the plastic deformation of workpiece material at higher temperature resulted in the increase in surface roughness [32]. In addition, with the grinding depth of 0.02 mm, a higher grinding speed produced smaller-diameter chips, and each area of the grinding wheel surface participated in the machining process more frequently, which accelerated the clogging of grinding wheel, resulting in increased grinding power and heat generation [33]. When the grinding depth increased to 0.03 mm, the surface roughness was deteriorated and increased to ~0.60 μm. Obviously, the increase in grinding depth produced a higher level of grinding force and grinding temperature and the material was squeezed by abrasive particles and flowed plastically to both sides, resulting in the increase in surface roughness [34]. At a grinding depth of 0.02 mm when the feed speed increased from 0.26 m/s to 0.35 m/s, and at the grinding depth of 0.03 mm when the feed speed increased from 0.19 m/s to 0.26 m/s, the decrease in Sa was less than 0.034 μm. However, at the feed speed of 0.19 m/s, the increase in Sa exceeded 0.097 μm when the grinding depth increased from 0.02 mm to 0.03 mm. The Sa decreased slightly when it increased from 0.26 m/s to 0.35 m/s, which might be caused by the squeezing effect; the influence on Sa was limited when compared with the effect of grinding depth. This is also consistent with the results of ANOVA in Table 4.

## 4. Conclusions

(1) The grinding temperature increased with the grinding depth and grinding speed, with the highest level of ~290 °C. The feed speed presented the most significant effect on grinding temperature with the corresponding percentage contribution ratio of 33.7%. Both work hardening and thermal softening were observed during grinding of quenched gear samples, and aggressive grinding parameters intensified the thermal softening effect, resulting in a reduction in microhardness within the subsurface layer.
(2) The thickness of the white layer exceeded 100 μm when the grinding depth increased to 0.03 mm and it could be subdivided into three different layers owing to the contribution of mechanical extrusion and the thermal effect. The top layer mainly featured plastic deformation and the second one was composed of fine-grained martensite. Coarse-grained acicular martensite was found at the interface between the white layer and the softened dark layer.
(3) The ground surface topography showed several scratches and typical grooves and some particles were embedded into the ground surface, which was probably caused by the shedding and breaking of abrasive particles. When the grinding depth increased to 0.03 mm, the grinding surface roughness (Sa) was relatively high and reached up to ~0.60 μm. The percentage contribution ratio of the grinding depth on surface roughness was higher than 50%, mainly owing to severe plastic deformation under grinding wheel extrusion and the thermal effect.

## Figures and Tables

**Table 1:** Chemical composition of 20Cr2Ni4A steel (mass fraction, %) [13].

C	Si	Mn	Cr	Ni	Fe	S
0.17–0.23	0.17–0.37	0.30–0.60	1.25–1.65	3.25–3.65	≥95.00	≤0.03

**Table 2:** Parameters set in the gear forming grinding experiment.

Feed Speed (m/s)	Grinding Speed (m/s)	Grinding Depth (mm)	Number of Passes
0.19	26	0.02	12
	30	0.02	12
		0.03	8
	34	0.02	12
		0.03	8
0.26	26	0.02	12
	30	0.02	12
		0.03	8
	34	0.02	12
		0.03	8
0.35	26	0.02	12
	30	0.02	12
	34	0.02	12

**Table 3:** ANOVA of grinding temperature at different parameters.

Source	DOE	Adj SS	Adj MS	F	PCR
Feed speed (m/s)	2	14,512	7255.8	8.22 *	33.7%
Grinding speed (m/s)	2	2975	1487.5	1.69	3.2%
Grinding depth (mm)	1	5794	5793.6	6.56 *	13.0%
Error	7	6178	882.5		50.1%
Total	12	37,832			100%

* Significant at the 5% level, F_0.05, 2, 7_ = 4.74, F_0.05, 1, 7_ = 5.59.

**Table 4:** ANOVA for surface roughness (Sa).

Source	DOE	Adj SS	Adj MS	F	PCR
Feed speed (m/s)	2	0.002239	0.001120	2.06	5.1%
Grinding speed (m/s)	2	0.000652	0.000326	0.60	0%
Grinding depth (mm)	1	0.012133	0.012133	22.35 *	51.0%
Error	7	0.003801	0.000543		43.9%
Total	12	0.022745			100%

* Significant at the 5% level, F_0.05, 2, 7_ = 4.74, F_0.05, 1, 7_ = 5.59.

## Data Availability

The data that support the findings of this study are available from the corresponding author upon reasonable request.
